# Use of smart patches by athletes: A concise SWOT analysis

**DOI:** 10.3389/fphys.2023.1055173

**Published:** 2023-03-22

**Authors:** Nina Verdel, Klas Hjort, Billy Sperlich, Hans-Christer Holmberg, Matej Supej

**Affiliations:** ^1^ Swedish Winter Sports Research Centre, Mid Sweden University, Sundsvall, Sweden; ^2^ Faculty of Sport, University of Ljubljana, Ljubljana, Slovenia; ^3^ Department of Materials Science and Engineering, Uppsala University, Uppsala, Sweden; ^4^ Integrative and Experimental Exercise Science and Training, Institute of Sport Science, University of Würzburg, Würzburg, Germany; ^5^ Department of Health Sciences, Luleå University of Technology, Luleå, Sweden; ^6^ Department of Physiology and Pharmacology, Biomedicum C5, Karolinska Institutet, Stockholm, Sweden

**Keywords:** sensor, sport, internet of thing (IoT), wearable, strechable, flexible, electronis, IMU

## Introduction

Recent advances in wearable technology have produced a variety of devices worn directly on or attached loosely to the body, which can be used to monitor a variety of parameters in athletes during training and competition—including patterns of movement (Loader et al., 2012), workload (Mooney et al., 2011) and biometric markers (Foster et al., 2010)—in a manner that is increasingly accurate and affordable. In addition to helping to maximize performance, this information allows for the identification of biomechanical, thermal, neuro-muscular of metabolic fatigue and thereby helps prevent injury (Li et al., 2016). However, some of the wearables currently available are still bulky and/or uncomfortable to wear, and their fixation to the body is often unstable, affecting the accuracy of measurements and sometimes even leading to complete loss of data, especially in connection with intense movements (Düking et al., 2020).

These shortcomings may be overcome by the most recent wearable technology, i.e., smart patches that can be attached tightly to the body (Lee et al., 2016; Ortega et al., 2019; Kim et al., 2021). Smart patches, a type of wearable sensor, are used for diagnosis and long-term monitoring of the vital signs of patients, as well as, more recently, for monitoring athletic performance. These patches, which are attached to the body with a skin adhesive, can contain numerous sensors. Smart patches that fit tightly to the body provide fewer artefacts during measurements, which means that measured parameters are less dependent on algorithms. In addition, the new patches also enable metabolic sensing from sweat (Anastasova et al., 2017; Wiorek et al., 2020; Ghaffari et al., 2021) or minimally invasive sampled interstitial fluid (Veiseh and Langer, 2015; De la Paz et al., 2021). These devices are becoming increasingly versatile and, thanks to miniaturization, comfortable, as well as more sustainable for long-term use.

Here, we summarize our own experience concerning the strengths and weaknesses, as well as the opportunities and threats associated with the use of smart patches to monitor athletic performance, which can hopefully help improve the design of these devices for use in this context.

The primary SWOT matrix employed is illustrated in Table 1.

**TABLE 1:** A summary of the strengths and weaknesses, as well as opportunities and threats associated with the use of smart patches by athletes.

Strength	Weakness
• Low weight and small size, allowing comfortable and	• Low battery capacity
• unobtrusive use under the racing suit Wireless communication	• More difficult to attach than bands or straps
• Long-term monitoring	• No display of the data collected
• Adhesive fixation, ensuring reliability	• Unfashionable design showering
• Low cost	• Decreased adhesion due to sweat, Painful to remove the patches
• Flexibility and stretchability	• Airport security
• Simultaneous monitoring of physiological and biomechanical parameters in real-time	• Expensive subscriptions

Opportunities	Threats
• The capacity to monitor several locations on the body at the same time	• Potential irritation of the skin or allergic reaction
• The potential usage of Fat-IBC for communication between different smart patches	• Problems associated with wireless communication including potential loss of data, relatively slow sampling rates, and ethical issues about the security of the data collected (e.ge-doping)
• The ability to monitor both during competitions and for research purposes, both outdoors and indoors, under realistic conditions	• Potential problems with both software and hardware
• Protentional connection to the Internet of Things Provision of real-time information to spectators	• Data reliance
• Protentional connection to the Internet of Things Provision of real-time information to spectators	• Unreliable or no valid data
• Tattoo”/colour appearance of patches	

## Strengths

The small size and low weight of smart patches make them less obtrusive than other wearables, so athletes can comfortably wear them under their race suits, during training, competitions as well as for research purposes and, of course, under their everyday clothes. In addition, since smart patches transmit the information they collect wirelessly, athletes can wear them while sleeping, during daily activities, etc., which allows long-term and 24-h monitoring (Sperlich and Holmberg, 2017). Longitudinal monitoring with high-resolution data can be particularly valuable in connection with both determining whether an athlete is adapting as desired to the training program and minimizing the risk of non-functional overuse (fatigue lasting for weeks to months), injury, and illness (Halson 2014; Sperlich and Holmberg, 2017).

The adhesive attachment of smart patches, which prevents them from moving, ensures high-quality biomechanical signals. At the same time, the relatively low price of smart patches combined with their wireless communication allows simultaneous monitoring and comparison of multiple in real-time.

Since many smart patches are flexible and stretchable, they can be attached at different locations on the body where other wearable sensors cannot, e.g., the bone in the heel. Moreover, if they are synchronized in time and arranged to communicate with one another, smart patches can be used to monitor both biomechanical and physiological parameters, including ECG signals, heart rate, temperature, and metabolic biomarkers (e.g., glucose, lactate, pyruvate, pH) at the same time.

## Weaknesses

Striving for a small size of electronic hardware and battery to make the smart patches unobtrusive means that the small battery must be charged or replaced more frequently than in the case of, e.g., smartwatches. Although smart patches can be worn at locations on the body where other wearable sensors cannot be attached, proper attachment of smart patches can be more difficult than the utilization of other wearables such as sports watches, wristbands, bracelets, etc. For example, an athlete might find it quite difficult to apply an adhesive smart patch to his/her forearm properly using only the other hand, since, with only one hand, the adhesive section might stick to itself. In addition, when there is considerable moisture (e.g., if the athlete sweats profusely, goes out in the rain or takes a shower), the smart patch may peel off. On the other hand, removing the patch can be painful if the smart patch adheres very well.

Another potential weakness is that because of their small size, smart patches do not display the information they gather, i.e., an athlete cannot see his/her running speed, for example, in real-time. However, it should be relatively easy to connect a smart patch to another device that can display data in real-time. In addition, long-term monitoring may be affected by airport security checks when athletes travel to competitions or training camps, as they may need to remove the patches.

The design of many smartwatches and wristbands (such as Garmin, Whoop, Suunto, etc.) is quite fashionable, making them popular to wear throughout the day (Juhlin et al., 2016). The less fashionable design of smart patches might be problematic for some users, e.g., during business meetings or other non-athletic activities, although in that case, these devices can most often be hidden under one´s clothing.

Moreover, using the smart patches and transferring the data to the smart device/cloud requires the use of an app from the manufacturer, and the subscription could become costly over time.

## Opportunities

Smart patches can be attached at several different locations at the same time, e.g., the right and left legs and the trunk, which can provide biomechanical information concerning potential asymmetry between the left and right sides of the body (Mitchell et al., 2015; Ueberschär et al., 2019). For monitoring asymmetries of the body, all of the sensors must be synchronized (Mitchell et al., 2015), which requires communication between all of the smart patches attached. Appropriate communication between different smart patches can be achieved, e.g., with intra-body communication, e.g., Fat-IBC, (Asan, 2019; Asan et al., 2021), rather than by Bluetooth.

In addition, one of the opportunities is compliance, i.e., athletes do not have to worry about patch charging, data transmission, etc., and can focus more on training and recovery.

Moreover, asymmetries and all other parameters can be monitored both during competitions and for research purposes, both outdoors and indoors, under realistic conditions. In addition, the smart patches can be linked to the Internet of Things (IoT), which would enable the provision of live information during competitions to coaches and/or spectators.

As mentioned above the design of smart patches is less fashionable, however, further, development could bring beautiful colorful or even tattoo-looking patches (KaoCindy et al., 2016).

## Threats

Smart patches are adhered to the skin with an adhesive that may cause irritation or even allergic reactions in more sensitive skin.

A small size of smart patches may not allow for internal storage of data. For long-term monitoring with high frequency, the device requires considerable internal memory, which means that it must be larger. To avoid this increase in size, data must be transferred wirelessly (e.g., through Bluetooth) to the cloud or a mobile device or computer, resulting in slower sampling rates (and thereby poorer accuracy or even an inability to monitor certain parameters) than is the case with gold standard devices. For example, the asymmetry in ground contact time (GCT) for healthy runners is typically less than 3% (Stiffler-Joachim et al., 2021) and the GCT for elite female runners at a speed of 5 m/s is approximately 150 m (Chapman et al., 2012). Consequently, in order to detect differences in GCT asymmetry during running of <4.5 m, data must be acquired at a minimum frequency of approximately 250 Hz, which is unobtainable with most smartwatches presently on the market.

In addition, if not handled correctly, wireless communication involves both the possibility of complete loss of data for some period of time as well as security issues (Austen, 2015; Düking et al., 2017; Wearable Sensor Technology for Monitoring Training Load and Health in the Athletic Populatio**n** | Frontiers Research Topic), such as the theft or modification of data (which could result in e-doping). Such possibilities could lead to the use of smart patches being banned in connection with competitions.

Since smart patches have come onto the market relatively recently, problems with both their hardware and software (Jiang et al., 2015) can be expected and after-sales support and service require further improvement. In addition, new wearables (e.g., smart patches) are unfortunately often marketed with exaggerated claims that lack a solid scientific basis, so the data they provide may be unreliable or not valid. Consequently, relying on data provided by smart patches can be unfavorable or even harmful for athletes.

## Summary

The use of smart patches by athletes is associated with a variety of strengths, opportunities, weaknesses, and threats, Table 1.

In summary, although smart patches currently still have many disadvantages, these are already outweighed by their advantages in connection with a variety of applications, e.g., monitoring of ECG (SmartCardia, 2023), testing hydration (Gx App For Sports Science & Recovery, 2023), detection of exposure of the skin to UV light (Patch, 2023), etc. Their most impressive strengths include comfort, due to their lightweight and small size; the ability to monitor both biochemical and physiological parameters at several different locations on the body simultaneously, as well as wireless communication, which enables long-term monitoring that improves reliability, and low cost. Accordingly, in connection with the applications listed above, as well as others, smart patches are already preferable to other wearables. At the same time, several weaknesses of these devices **--** including low battery capacity, difficult attachment compared to bands and straps, potential problems with both software and hardware, lack of real-time display, decreased adhesion due to sweat, painful removal of the patches, unfashionable design, and expensive subscriptions—need to be addressed through research and industrial development. Among the most important improvements required are greater battery capacity, better wireless transmission that allows more rapid sampling while minimizing loss of data, and less irritating adhesion that is not comprised by wet conditions.
